# Sympatric Yaks and Plateau Pikas Promote Microbial Diversity and Similarity by the Mutual Utilization of Gut Microbiota

**DOI:** 10.3390/microorganisms9091890

**Published:** 2021-09-06

**Authors:** Haibo Fu, Liangzhi Zhang, Chao Fan, Wenjing Li, Chuanfa Liu, He Zhang, Qi Cheng, Yanming Zhang

**Affiliations:** 1 Key Laboratory of Adaptation and Evolution of Plateau Biota, Northwest Institute of Plateau Biology, Chinese Academy of Sciences, Xining 810008, China; fuhb@nwipb.cas.cn (H.F.); lzzhang@nwipb.cas.cn (L.Z.); fanchao@nwipb.cas.cn (C.F.); wjli@nwipb.cas.cn (W.L.); liuchuanfa15@mails.ucas.ac.cn (C.L.); zhanghe072@163.com (H.Z.); cq19900924@sina.cn (Q.C.); 2 Qinghai Key Laboratory of Animal Ecological Genomics, Northwest Institute of Plateau Biology, Chinese Academy of Sciences, Xining 810008, China; 3 University of Chinese Academy of Sciences, Beijing 100049, China

**Keywords:** gut microbiota, plateau pika, yak, horizontal transmission, reciprocity

## Abstract

Interactions between species provide the basis for understanding coexisting mechanisms. The plateau pika (*Ochotona curzoniae*) and the yak (*Bos grunniens*) are considered competitors because they have shared habitats and consumed similar food on the Qinghai–Tibetan Plateau for more than 1 million years. Interestingly, the population density of plateau pikas increases with yak population expansion and subsequent overgrazing. To reveal the underlying mechanism, we sequenced the fecal microbial 16S rDNA from both sympatric and allopatric pikas and yaks. Our results indicated that sympatry increased both gut microbial diversity and similarity between pikas and yaks. The abundance of Firmicutes, Proteobacteria, Cyanobacteria, and Tenericutes decreased, while that of Verrucomicrobia increased in sympatric pikas. As for sympatric yaks, Firmicutes, Bacteroidetes, and Spirochaetes significantly increased, while Cyanobacteria, Euryarchaeota, and Verrucomicrobia significantly decreased. In sympatry, plateau pikas acquired 2692 OTUs from yaks, and yaks obtained 453 OTUs from pikas. The predominant horizontally transmitted bacteria were Firmicutes, Bacteroidetes, Verrucomicrobia, and Proteobacteria. These bacteria enhanced the enrichment of pathways related to prebiotics and immunity for pikas, such as heparin sulfate, heparin, chitin disaccharide, chondroitin-sulfate-ABC, and chondroitin-AC degradation pathways. In yaks, the horizontally transmitted bacteria enhanced pathways related to hepatoprotection, xenobiotic biodegradation, and detoxification. Our results suggest that horizontal transmission is a process of selection, and pikas and yaks tend to develop reciprocity through the horizontal transmission of gut microbiota.

## 1. Introduction

In nature, organisms coexist through complex interspecific interactions, such as competition, predation, commensalism, and mutualism, which contribute to the biodiversity and stability of biocoenosis [1,2]. Traditional studies have generally assumed that sympatric herbivores are competitors because they consume similar plants and share habitats, leading to a high overlap of diet and space niche [1,3]. However, recent studies have provided considerably different insights. For example, livestock promotes outbreaks of sympatric locusts *(Oedaleus asiaticus)* under heavy grazing conditions [4]. Furthermore, wild ungulates positively influence cattle by enhancing the dietary crude protein content during the wet season, while the opposite interaction occurs during the dry season [5]. These studies have indicated that environmental factors may modify interspecific interactions and initiate a shift from competition to reciprocity. In particular, the gut microbiota, which participates in the biosynthesis of vitamins, cellulose degradation, and the development of immunity against disease, enhances the host’s food digestion and nutrition utilization [6,7,8]. For instance, gut microbiota that originates from pets enhances the health of babies by lowering the rates of asthma [9]. Furthermore, many animals acquire microbes from their environment or other species [10], and horizontally transmitted bacteria confer resistance to natural enemies to the host [11] and help their host overcome extreme environmental stress [12]. These examples indicate that animals can be beneficial to each other through the horizontal transmission of gut microbiota, which may also be a strong mediating factor between hosts. Overall, this highlights the need to explore interspecific interactions by investigating the gut microbiota.

As a typical herbivorous species on the Qinghai–Tibetan Plateau (QTP), yak speciated in northeastern Eurasia (i.e., northern China, Inner Mongolia, eastern Siberia, and northern mid-Asia) 2.5 million years ago (Ma) and possess excellent tolerance of cold and hypoxic conditions, which has allowed this species to have a widespread distribution [13]. Likewise, as an indigenous mammal on the QTP, the plateau pika also prefers cold and hypoxic environments [14] and has been widely distributed on the QTP since 3.4 Ma [15,16]. These two species have coexisted for about 2.4 million years, and they are assumed to compete for scarce or limited plant foods and overlapping spaces [3]. However, with the rapid population growth of yaks in the last 6–7 decades, the density of plateau pikas has considerably increased [17,18]. Moreover, plateau pikas prefer to live on grasslands degraded by livestock overgrazing and are characterized by extremely low winter mortality [19]. Notably, there are two conventional interpretations of this phenomenon. A possible explanation is that livestock overgrazing lowers the height of vegetation and, hence, widens the field of vision of plateau pikas, which is advantageous for detecting predators [19,20]. Alternatively, another interpretation is that the stronger tolerance to toxic plant secondary metabolites (PSMs) of plateau pikas, compared with that of other herbivores, results in its outbreak in degraded regions, which are widely colonized by poisonous plants [21,22,23]. However, we have found that plateau pikas survive in winter by eating yak feces when food is short, and horizontal transmission of gut microbiota occurred during this process [24]; these scenes made us question if pikas eat yak feces in summer, or if horizontal transmission of gut microbiota also occurred in summer.

Based on the above scenes, plateau pikas and yaks may not only be simple competitors, but their relationship may also be characterized by reciprocity. However, more evidence is required to fill this knowledge gap. Therefore, in this study, we evaluated the sympatric pikas, yaks, allopatric pikas, and allopatric yaks to identify the sympatric effects on the gut microbiota of pikas and yaks. We collected fecal samples of pikas and yaks, sequenced the 16S rDNA of gut microbiota, and predicted microbial function to assess their implication. We determined whether sympatry contributed to the horizontal transmission of gut microbiota and diversity between pikas and yaks and appraised the gut microbial functional implications and benefits of sympatric effects on the host.

## 2. Materials and Methods

### 2.1. Experimental Procedures

**Figure 1:**
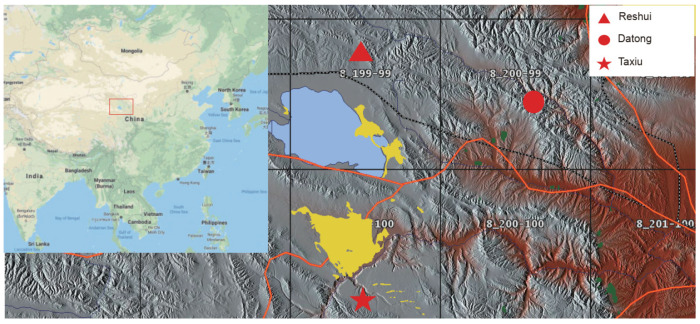
Map for fecal sample collection sites. Triangle denotes the sympatric plateau pika and yak collection sites. Circle denotes locations where allopatric yak’s fecal samples were collected. Pentagram denotes locations where allopatric plateau pika’s fecal samples were collected. The map of China comes from Google earth, and the map of Qinghai Province comes from an open-source website (https://maps-for-free.com/ (accessed on 8 February 2021)).

To assess horizontally transmitted bacteria, we designed a group of experiments based on previously described methods [25]. Three different sites, including sympatric pikas and yaks, allopatric pikas, and allopatric yaks, were identified. Samples from sympatric pikas and yaks were collected in the Reshui village, Qinghai, China (altitude: 3720 m; N: 37°9′39″, E: 100°28′40″; Figure 1), and the average annual temperature was about 2 °C [26]. To assess sympatry, we selected grasslands where only plateau pikas and yaks lived together, that is, without sheep, horses, and other animals. After investigating several sites, we found that Datong (i.e., the Datong Yak Farm in Qinghai Province; Figure 1) was ideal for evaluating allopatric yaks, as there were no Tibetan sheep, horses, or any other livestock in that region, and the altitude of Datong is 2700–3000 m, while the habitat of plateau pikas is above 3000 m [14]. Therefore, yaks in the farm developed their own gut microbiota, without bacteria from plateau pikas or other livestock. The ecosystem and vegetation types in the farm were similar to those at the sympatric site. In contrast, we searched for pika-specific habitats but failed to find a separate distribution site for pikas. As a consequence, we constructed steel enclosures (30 m × 50 m) that served as isolated distribution sites for the plateau pikas in Taxiu Town, Guinan County, Qinghai Province (altitude: 3360 m; N: 35°30′31″, E: 100°39′39″; Figure 1), where pikas were maintained in geographic isolation and without contact with yaks or other animals. To prevent the pikas escaping, the steel enclosures were buried 50 cm underground and 1 m above ground [27]. Notably, the ecosystem and vegetation types in the enclosures were similar to those at the sympatric site. Thus, the plateau pikas in the enclosure developed their own gut microbiota without bacteria from yaks or other animals.

### 2.2. Sample Collection

Plateau pikas that lived in sympatry with yaks were trapped using the live-trapping method [28], kept in covered cages that had been sterilized using 75% alcohol, and their feces were collected as soon as they were available. At the same sites, the fresh feces of sympatric yaks were collected in 10 mL centrifuge tubes. All fecal samples were stored in liquid nitrogen. 

All of the samples were collected in summer (sympatric sample: July; allopatric sample: August), and all of the samples were from adult individuals. During the sampling of sympatric yaks, we had at least four people to stare at the yaks; we would drive away the yak after we collected one’s feces. For the sympatric pikas, we put them in separate cages; there was only one pika in each cage. For the allopatric yaks and pikas, we followed the same operation as the sympatric samples. 

The enclosure of allopatric was surrounded by a closed farm; no yaks, sheep, horses and other animals could enter the farm, and certainly, no animals could enter the enclosure. There were no pikas or other animals lived near the site of enclosure, as the experiment was elaborately designed; we checked the place before it was used as an experimental site. Additionally, the pikas in the enclosure were born in the enclosure; they never left the enclosure. The gut microbiota of allopatric pikas and allopatric yaks were used as references to filter horizontally transmitted bacteria. For conciseness, we defined a series of abbreviations for different group names. The PA represents the pikas in the allopatric enclosure, PS represents sympatric pikas with yaks, YS represents sympatric yaks with pikas, and YA represents yaks in allopatric farms. In total, 43 fecal samples were collected (Table S1).

### 2.3. DNA Extraction and Sequencing

In total, we extracted the DNA following the practicable procedures in our previous reports [29,30]. Microbial DNA was extracted using a TruSeq DNA PCR-Free Sample Preparation Kit (Illumina, San Diego, CA, USA). The V3 and V4 regions of 16S rDNA were amplifiedbased on the primers 341F (5′-CCTAYGGGRBGCASCAG-3′) and 806R (5′-GGACTACNNGGGTATCTAAT-3′) [31,32]. The PCR products were quantified and purified with a fluorometer (QuantiFluor; Promega, Madison, WI, USA) before being sequenced on the HiSeq 2500 platform with the PE250 model (Illumina, San Diego, CA, USA).

### 2.4. 16S rDNA Data Analysis

According to the previous reports [29,30], the paired reads were merged into a single tag based on the overlapping region between each paired read using FLASH software (v 1.2.11), with a minimum 10 bp overlap and 20% mismatch allowed in the overlapping region. Subsequently, we conducted filtering operations according to the procedures provided by QIIME 1.9.1 [33]. The details were as follows: the sequences with a cut-off at Q-value > 20, N-base > 10% and the sequences which were shorter than 300 bp were trimmed. After that, we aligned the clean tags against the GOLD database (version microbiomeutil-r20110519) based on the UCHIME algorithm to discard the chimers and singletons, and finally obtained the effective tags.

The effective tags were further used to search against the microbial reference database of Greengenes 13_8 [34] and those tags were clustered into OTUs based on the standard of 97% identity with the UCLUST algorithm. The representative OTUs were classified using Pynast, and the taxonomy of the OTUs was assigned using the UCLUST algorithm. The mitochondria, chloroplasts, and unclassified OTUs at the kingdom level were excluded from the OTU table. Diversity indexes were computed using alpha_diversity.py and beta_diversity.py in the QIIME pipeline. Significant differences in alpha diversity and Bray–Curtis dissimilarity were detected using the Mann–Whitney U test. Finally, we measured the allopatric and sympatric effects on the gut microbial composition of pikas and yaks using UniFrac distance. 

### 2.5. Identification of Horizontally Transmitted Microbiota

We identified horizontally transmitted OTUs by comparing the gut microbiota of yaks and plateau pikas following the methods described in a previous study [25]. The OTUs horizontally transmitted from yaks were shared by all the plateau pikas except the pikas in the enclosures, which had not been in contact with yaks. Likewise, the OTUs horizontally transmitted from pikas were found in all the yaks except the one in Datong farm, which lived without pikas. To evaluate the profile of microbes shared between sympatric heterospecifics, we displayed the number and composition of horizontally transmitted OTUs in Venn diagrams using R v3.2.2 and GraphPad Prism v6.00.

### 2.6. Function Prediction and Comparison 

To measure the effects of horizontally transmitted microbes on their new hosts and identify pathways that were significantly enhanced by horizontally transmitted OTUs, we compared the functional pathways of host OTUs (i.e., excluding the horizontally transmitted OTUs) to the total OTUs (i.e., including the horizontally transmitted OTUs) in pikas and yaks. The enzyme pathways were predicted using PICRUSt2 v2.3.0 [35]. 

## 3. Results

### 3.1. Differences in Gut Microbial Communities between Plateau Pikas and Yaks

**Figure 2:**
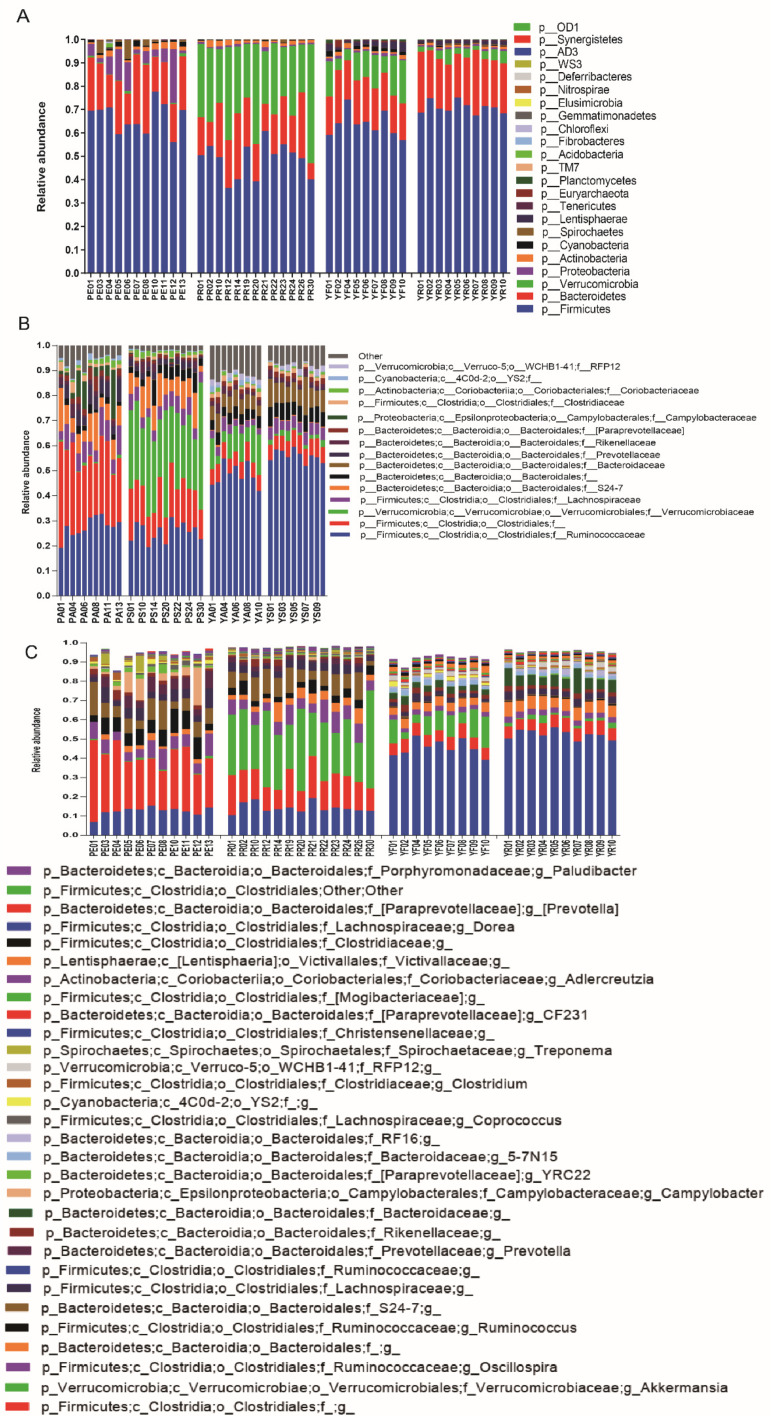
Composition of gut microbiota of plateau pikas and yaks in sympatry and allopatry. The “PA”: the pikas in the allopatric enclosure, “PS”: sympatric pikas with yaks, “YS”: sympatric yaks with pikas, and “YA”: yaks in allopatric farms. (**A**) Abundance of gut microbiota at phylum level. (**B**) Top 15 abundant gut microbiota at family level. (**C**) Top 30 abundant gut microbiota at genus level.

**Figure 3:**
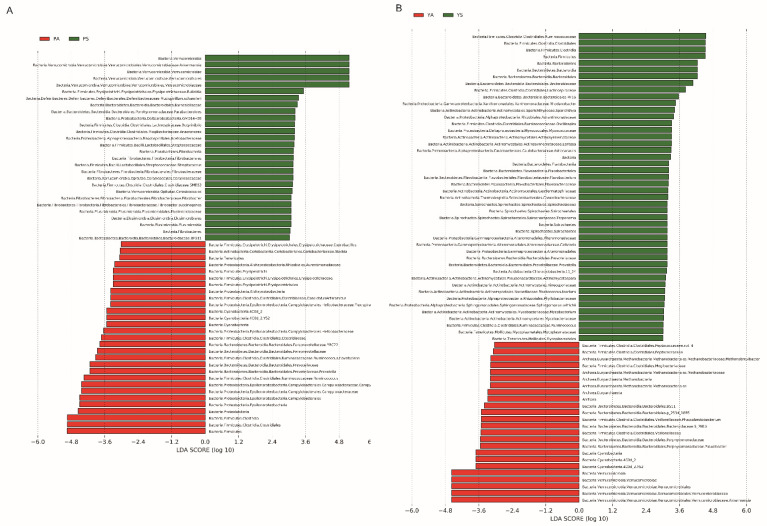
(**A**) Biomarkers of gut microbiota among different pika’s population in Lefse result. (**B**) Biomarkers of gut microbiota among different yak’s population in Lefse result. The “PA”: the pikas in the allopatric enclosure, “PS”: sympatric pikas with yaks, “YS”: sympatric yaks with pikas, and “YA”: yaks in allopatric farms.

In total, we obtained 3,439,015 16S rDNA sequences from 43 samples (Table S1). Subsequently, the sequences were classified into 8887 operational taxonomic units (OTUs) with a 97% identity cut-off. The gut microbiotas of pikas and yaks were dominated at the phylum level by Firmicutes, Bacteroidetes, Verrucomicrobia, Proteobacteria, and Actinobacteria, which accounted for over 90% of the total communities (Figure 2A; Table S2). At the family level, Ruminococcaceae, o__Clostridiales;f__, f__Verrucomicrobiaceae, and f__Lachnospiraceae were the most abundant (Figure 2B). Further analysis of the 30 most abundant genera revealed a marked variation in gut microbiota between sympatric individuals and their allopatric conspecifics (Figure 2C). When comparing the allopatric pikas to the pikas sympatric with yaks, elevated abundances of Firmicutes, Proteobacteria, Cyanobacteria, and Tenericutes were observed in allopatric pikas (Figure 2C; Table S2), whereas Verrucomicrobia were more abundant in sympatric pikas (Figure 3A; Table S2). The response of the gut microbiota of yaks to sympatry was different from that of sympatric pika. In other words, the abundances of Firmicutes, Bacteroidetes, and Spirochaetes were significantly greater in sympatric yaks (Figure 3B; Table S2), while Cyanobacteria, Euryarchaeota, and Verrucomicrobia were significantly more abundant in the allopatric yaks (Figure 3B; Table S2). At the genus level, allopatry in pikas induced significant increases in g_*Campylobacter*, g_*Prevotella*, g_*Ruminococcus*, and g_YRC22 (Figure 3A; Table S2), whereas there was a decrease in *g_Akkermansia* in allopatric pikas (Figure 3A; Table S2). Moreover, *g_Akkermansia* was more abundant in allopatric yaks (Figure 3B)**.**

### 3.2. Elevated Microbial Diversity in Sympatry 

**Figure 4:**
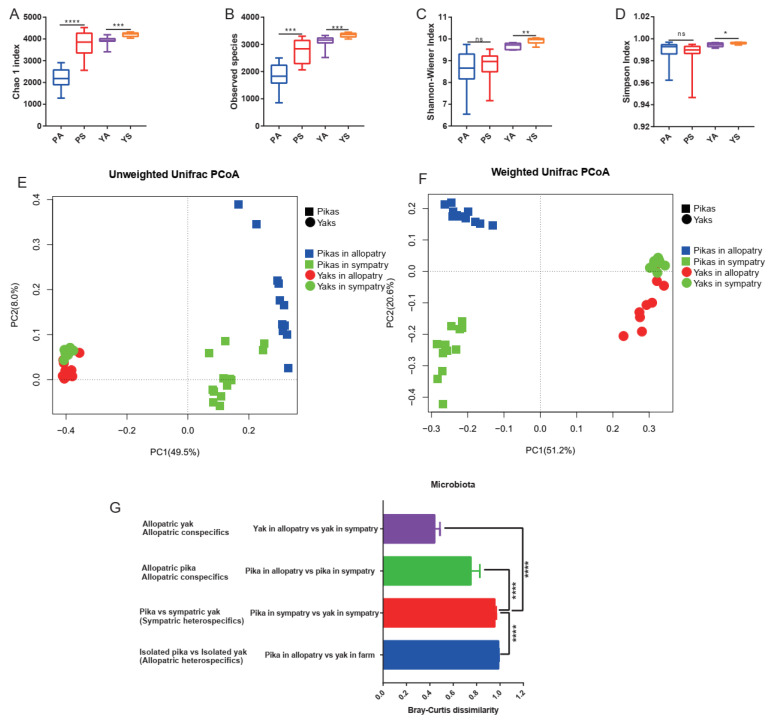
(**A**) The Chao1 index with comparison between allopatric groups and sympatric groups. The “PA”: the pikas in the allopatric enclosure, “PS”: sympatric pikas with yaks, “YS”: sympatric yaks with pikas, and “YA”: yaks in allopatric farms. (**B**) The observed species number with comparison between allopatric groups and sympatric groups. (**C**) The Shannon diversity index with comparison between allopatric groups and sympatric groups. (**D**) The Simpson index with comparison between allopatric groups and sympatric groups. (**E**) Unweighted UniFrac distance across allopatric individuals and sympatric individuals. (**F**) Weighted UniFrac distance across allopatric individuals and sympatric individuals. (**G**) Bray–Curtis dissimilarities with comparison among sympatric congenerics, allopatric congenerics, sympatric heterogenerics, and allopatric congenerics.

Both allopatric pikas and allopatric yaks had a lower Chao1 index and observed species number than their sympatric conspecifics (Figure 4A,B). As for the Shannon–Wiener and Simpson indices, allopatric pikas were still characterized by lower values, although without significant variations (Figure 4C,D), and allopatric yaks displayed significantly lower values (Figure 4C,D). Notably, sympatric pikas and yaks clustered more closely than allopatric populations (Figure 4E,F). Furthermore, the gut microbial composition based on the Bray–Curtis distance revealed that the microbial communities in conspecific individuals were more similar to each other than to the microbial communities of heterospecific individuals, even if conspecific individuals did not share the same environment or habitat (Figure 4G; Mann–Whitney U-test, U = 40, *p* < 0.0001). In addition, a convergence of the microbial communities was observed between sympatric heterospecifics compared with the allopatric populations (Figure 4G), which indicated an increased sharing of gut microbes between sympatric heterospecifics (Mann–Whitney U-test, U = 0, *p* < 0.0001).

### 3.3. Horizontally Transmitted OTU Clusters Shared between Plateau Pikas and Yaks

**Figure 5:**
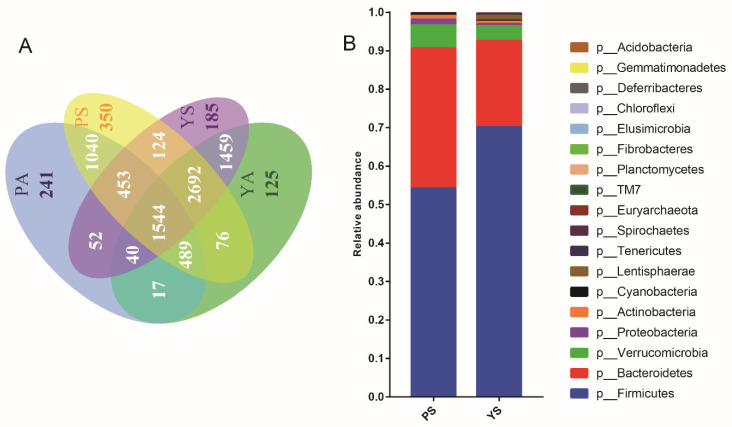
The horizontal transmitted OTU cluster number between the sympatric pikas and yaks. The “PA”: the pikas in the allopatric enclosure, “PS”: sympatric pikas with yaks, “YS”: sympatric yaks with pikas, and “YA”: yaks in allopatric farms. (**A**) The section labeled 2692 denotes those OTU clusters which plateau pika acquired from sympatric yak; the section labeled 453 denotes those OTU clusters which yak acquired from the sympatric plateau pika. (**B**) Relative abundance of horizontal transmitted bacteria at phylum level in sympatric pikas and yaks.

In total, 4813 (56.60%) OTUs were shared by sympatric pikas and yaks (Figure 5A). However, only 2090 (25.22%) OTUs were shared between allopatric pikas and yaks (Figure 5A). Notably, the prevalence of OTUs shared between sympatric heterospecifics often indicates the horizontal transmission of gut microbes [25].

The OTU clusters that were simultaneously present in sympatric pikas and yaks and also in allopatric yaks, but absent in allopatric pikas, were considered to be horizontally transmitted from yaks to pikas, since their presence depended on yaks and were only detected in pikas sympatric with yaks. Conversely, the OTU clusters that were simultaneously present in sympatric pikas, yaks, and allopatric pikas, but not in allopatric yaks, were defined as horizontally transmitted from pikas to yaks, since their presence depended on that of pikas and could only be identified in yaks in sympatry with pikas. Notably, pikas obtained more bacterial taxa from yaks than yaks obtained bacterial taxa from pikas (i.e., 2692 and 453 OTUs, respectively) (Figure 5A). Furthermore, these horizontally transmitted bacterial taxa spanned 18 phyla and 185 genera (Tables S3 and S4), and the most dominant phyla were Firmicutes, Bacteroidetes, Verrucomicrobia, and Proteobacteria (Figure 5B; Table S3). The horizontal transmission of the gut microbiota was imbalanced between the pikas and yaks. Bacteroidetes, Verrucomicrobia, and Proteobacteria tended to be transmitted to pikas from yaks, while Firmicutes tended to be transmitted to yaks from pikas (Figure 5B).

### 3.4. Functional Effects of Horizontally Transmitted Bacteria on Their New Hosts

The pathways from horizontally transmitted microbes mainly improved the degradation of polysaccharides through enzymes such as heparin-sulfate lyase (EC: 4.2.2.8), heparin lyase (EC: 4.2.2.7), chitin disaccharide deacetylase (EC: 3.5.1.105), chondroitin-sulfate-ABC endolyase (EC: 4.2.2.20 and EC: 4.2.2.21), and chondroitin AC lyase (EC: 4.2.2.5) (Figure S1A; Table S5).

Yaks obtained bacteria associated with hepatoprotection, xenobiotic biodegradation, and detoxification by mycothiol biosynthesis (Figure S1B; Table S5). The hepatoprotective pathways included numerous enzymes, namely betaine-aldehyde dehydrogenase (EC: 1.2.1.8), malonate-semialdehyde dehydrogenase (EC: 1.2.1.18), methylmalonate-semialdehyde dehydrogenase (CoA acylation; EC: 1.2.1.27), L-lactate dehydrogenase (cytochrome; EC: 1.1.2.3), 2-oxo-4-hydroxy-4-carboxy-5-ureidoimidazoline decarboxylase (EC: 4.1.1.9), allantoicase (EC: 3.5.3.4), and uronate dehydrogenase (EC: 1.1.1.203; Figure S1B; Table S5). The pathways of xenobiotic biodegradation included the following enzymes: alkanesulfonate monooxygenase (EC: 1.14.14.5), vanillate monooxygenase (EC: 1.14.13.82), 3-(3-hydroxy-phenyl) propanoic acid hydroxylase (EC: 1.14.13.127), (2Z,6E)-farnesyl diphosphate synthase (EC: 2.5.1.68), nicotine blue oxidoreductase (EC: 1.1.1.328), alkane 1-monooxygenase (EC: 1.14.15.3), 4-hydroxybenzoate 3-monooxygenase (EC: 1.14.13.2), allophanate hydrolase (EC: 3.5.1.54), formaldehyde dehydrogenase (EC: 1.2.1.46), 4-hydroxymandelate oxidase (EC: 1.1.3.46), 4-hydroxy-4-methyl-2-oxoglutarate aldolase (EC: 4.1.3.17), glycine dehydrogenase (cyanide-forming; EC: 1.4.99.5), 6-hydroxypseudooxynicotine (EC: 1.5.99.14), 5-exo-hydroxycamphor dehydrogenase (EC: 1.1.1.327), trans-feruloyl-CoA hydratase (EC: 4.2.1.101), aliphatic aldoxime dehydratase (EC: 4.99.1.5), microsomal epoxide hydrolase (EC: 3.3.2.9), 4-hydroxyacetophenone monooxygenase (EC: 1.14.13.84), and pentachlorophenol monooxygenase (EC: 1.14.13.50; Figure S1B; Table S5). Finally, multiple enzymes were also involved in detoxification pathways by mycothiol biosynthesis, namely N-acetyl-1-D-myo-inositol-2-amino-2-deoxy-alpha-D-glucopyranoside deacetylase (EC: 3.5.1.103), L-cysteine:1D-myo-inositol 2-amino-2-deoxy-alpha-D-glucopyranoside ligase (EC: 6.3.1.13), mycothiol S-conjugate amidase (EC: 3.5.1.115), mycothiol synthase (EC: 2.3.1.189), S-(hydroxymethyl)mycothiol dehydrogenase (EC: 1.1.1.306), mycoredoxin (EC: 1.20.4.3), L-histidine N(alpha)-methyltransferase (EC: 2.1.1.44), and gamma-glutamyl cysteine (EC: 1.14.99.50) (Figure S1B; Table S5).

### 3.5. Functional Differences of Gut Microbiota between the Allopatric and Sympatric Individuals

**Figure 6:**
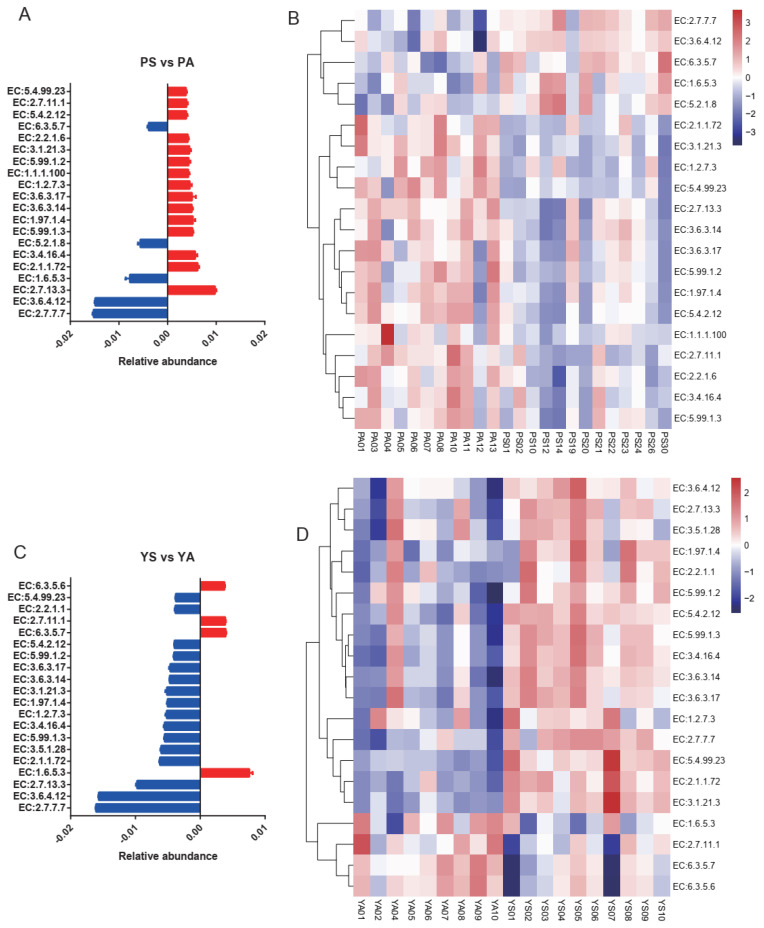
The top 20 enzymes with significant differences between sympatric groups and allopatric groups. The “PA”: the pikas in the allopatric enclosure, “PS”: sympatric pikas with yaks, “YS”: sympatric yaks with pikas, and “YA”: yaks in allopatric farms. (**A**,**B**) The enzymes with significant differences between sympatric pikas and allopatric pikas. (**C**,**D**) The enzymes with significant differences between sympatric yaks and allopatric yaks.

For pikas, the allopatric individuals had 15 enzymes higher than the sympatric individuals, while the sympatric individuals had only 5 enzymes higher than the allopatric individuals in the top 20 enzymes with significant differences (Figure 6A,B). Moreover, the allopatric yaks had only 4 enzymes higher than the sympatric yaks, while the sympatric yaks had 15 enzymes higher than the allopatric yaks in the top 20 enzymes, with significant differences (Figure 6C,D). An opposite trend of microbial enzymes with significant differences was observed between pikas and yaks.

## 4. Discussion

In our study, the dominant gut microbiota differed between allopatric and sympatric individuals, which indicates a strong influence of sympatry on the gut microbiota in heterospecifics. Notably, pikas and yaks have been considered as complete competitors in previous reports [1,3]. If this competition hypothesis provided an exact assessment between pikas and yaks, allopatric individuals would develop a lax gut microbiota with inefficient energy harvesting from food, as they would not need to cope with competitive pressure. Conversely, sympatric species can develop an efficient gut microbiota to improve their competitive ecological advantages by improving assimilation, as the gut microbiota plays an important role in energy absorption by decomposing the indigestible cellulose and hemicellulose for herbivores [36,37]. In our study, higher abundances of Firmicutes and lower abundances of Euryarchaeota and *Akkermansia* were observed in allopatric pikas than in sympatric pikas, highlighting an efficient gut microbial phenotype, since higher Firmicutes levels correspond to an excessive assimilation of energy in obese individuals [38]. Moreover, a lower abundance of Euryarchaeota resulted in a low level of methane emissions, as most Euryarchaeota are responsible for elevated methane yield [39,40]. In addition, *Akkermansia* contributes to energy expenditure in the host, and a higher *Akkermansia* abundance has been associated with a slim body [41,42]. Overall, we observed that allopatric pikas had multiple energy sources and reduced energy expenditure to maintain energy balance by reshaping the gut microbiota, thereby exhibiting an efficient gut microbial phenotype. Therefore, the conventional view on the relationship between pikas and yaks contradicts the results presented herein and does not account for the increase in the population density of pikas accompanied by the expansion of the yak population [17,18]. Together, these circumstances in the gut microbiota of sympatric pikas may result in a potential ecological benefit for the survival of sympatric pikas, even if they host inefficient gut microbiota. Furthermore, the variation in gut microbiota between sympatric and allopatric yaks was slight compared with that between pikas, and it was characterized by a trend opposite to that observed in pikas; for example, the abundance of Firmicutes was reduced, while that of *Akkermansia* was increased in allopatric yaks. This trend implies that the response mechanism of yaks to sympatry is different from that of pikas. Yaks sympatric with pikas tend to contain more immunity-related bacterial taxa, such as *Akkermansia*, which is associated with increased immunity and health [43].

Sympatry is associated with increased gut microbial diversity in both pikas and yaks, which implies a potential horizontal transmission between sympatric heterospecifics. It is possible that horizontal transmission significantly increases microbial diversity [44] and that the host acquires gut microbiota from other species to maintain a significantly greater species richness and biodiversity than non-gut environments [45,46]. Moreover, pikas eat the feces of yaks [24,47], which could increase the gut microbial diversity of pikas that live with yaks. Likewise, the soil-ingestion behavior of yaks may increase their gut microbial diversity when they live with pikas, as pikas excrete hard solid fecal pellets throughout the field [3,48]. Furthermore, the diversity of soil microbiota is increased by multiple animals living in sympatry, as these animals influence the soil structure and transfer microbes through fecal contamination or contact with each other [10,46,49]. In addition, plateau pikas and yaks can modify the plant community structure and increase diversity in the plant community by foraging, excavating, and grazing [50,51,52,53]. Accordingly, these changes in the plant community may increase the gut microbial diversity of pikas and yaks by providing a variety of diets. Moreover, the changes in the gut microbiota diversity indices reported in the present study indicated variations in comprehensive community structure rather than an increase in rare species, implying potential sympatry instead of allopatry. Notably, microbial diversity in the gut is regarded as a biomarker of health and metabolic capacity [54]. High gut microbial diversity often improves the host’s fitness by expanding the dietary niche in wild animals, as a diverse gut microbiota provides a comprehensive capacity to cope with the complex compounds in vegetable diet sources [55,56]. Therefore, individuals may expand their dietary niches through the horizontal transmission of the gut microbiota from their sympatric heterospecifics.

In the present study, sympatry also enhanced the similarity in the gut microbiota between pikas and yaks. Coprophagy and soil-ingestion behaviors, which are prevalent in pikas and yaks, respectively, cause a microbial transfer to new hosts through contact with feces or fecal-contaminated soil [57,58]. Likewise, convergence and increased similarity have also been observed in sympatric chimpanzees and gorillas (i.e., they shared more OTUs than they did in allopatric environments) due to the horizontal transmission of gut microbiota [25]. Horizontal transmission of gut microbiota between sympatric individuals may occur through incidental contact with feces or specific behavioral contact [59,60]. Furthermore, the horizontal transmission of gut microbiota, which leads to an increased similarity in gut microbiota, has also been reported in sympatric Malagasy mammals [59]. The observation of this phenomenon in different animals suggests that it may be common for sympatric animals to obtain bacteria through the horizontal transmission of gut microbiota.

In our study, noticeable inequalities of horizontal transmission were observed between the sympatric pikas and yaks, which indicates that the host species played an important role in the colonization of horizontally transmitted microbes. The horizontal transmission of bacteria can be affected by many factors, such as foraging, spatial activity patterns, or physical contact [25,61]. The fecal bacteria of yaks may be more accessible to sympatric pikas than the fecal bacteria of pikas for yaks, as pikas prefer to excrete their hard, solid balls of feces on the ground or in the grass, and the lush vegetation may prevent yaks from coming into contact with these feces. As a result, more bacteria were transferred from yaks to pikas than from pikas to yaks. Plateau pikas eat not only their own feces but also those of yaks [47]. Notably, a previous study linked the coprophagy of plateau pikas to the recycling of minerals and calories [47]. However, our study suggested that coprophagy might result in the acquisition of interspecific microbes and promote host adaptation to extreme environments.

Organisms often enhance their fitness by acquiring bacteria from other species or environments. For example, although coming from a lineage of carnivores, the giant panda possesses the ability to digest cellulose, acquired from horizontally transmitted microbes derived from bamboo [62]. Furthermore, it has been reported that the Japanese acquired carbohydrate-active enzymes from marine bacteria in seafood [63] and that bacteria horizontally transmitted from dogs to children reduce the risk of childhood allergic diseases [9]. In our study, the benefits of horizontally transmitted microbes in the new host differed across hosts. For pikas, horizontally transmitted microbes participate in prebiotic metabolism, and the corresponding prebiotics are composed of heteroglycans, including heparin, chitin, and chondroitin, which provide food for probiotics [64]. For example, *Lactobacillus* and *Bacillus*, which are horizontally transmitted from yaks to pikas (Table S5), could improve host immunity by neutralizing toxins [65]. Notably, strong immunity is essential for pikas, as they suffer from abundant parasitic infections, especially intestinal parasites [66]. Therefore, immune enhancement from horizontally transmitted microbes may contribute to the resistance of pikas to parasitic infections. Moreover, the plateau pika prefers plants that contain toxic PSMs, such as *Taraxacum sp., Plantago asiatica, Potentilla nivea*, and *Oxytropis sp.*; hence, it harbors the gut microbiota to degrade these toxic substances [21,22]. Generally, small mammals possess stronger resistance to PSMs than large mammals [67,68], a trait that is derived from the gut microbiota [55]. However, yaks and other large herbivorous mammals have an extremely weak capacity to cope with poisonous PSMs [67,68,69] and, hence, are often poisoned by accidentally foraging poisonous plants [70]. Therefore, yaks must employ alternative pathways to degrade PSMs, if possible. For instance, the genus *Rhodococcus*, which encodes multiple enzymes for the degradation of diverse toxic and xenobiotic compounds [71], was observed in a set of horizontally transmitted microbes from pikas to yaks. Previous studies have shown that horizontally transmitted microbes improve the tolerance to environmental stress in pea aphids (*Acyrthosiphon pisum*) [12], expand the host dietary niche [72], and decompose toxic compounds (creosote resin) in plants [73]. Likewise, the bacteria horizontally transmitted from pikas to yaks played important roles in hepatoprotection, xenobiotic biodegradation, and detoxification by mycothiol biosynthesis in yaks. These similar patterns in different animals highlight the point that animals may evolve an adaptive capacity for obtaining bacteria from their surrounding environment or other species, even from sympatric competitors, to cope with multiple challenges.

## 5. Conclusions

In summary, sympatric yaks and plateau pikas increased their ecological adaptation and obtained ecological advantages through the horizontal transmission of gut bacteria. That is, they enhanced their gut microbial diversity to expand their diet niche, which was mediated by horizontally transmitted bacteria. Additionally, they acquired horizontally transmitted bacteria that provided specific functional benefits to their hosts, such as intestinal immunity, hepatoprotection, xenobiotic biodegradation, and detoxification. Through horizontal transmission, these sympatric species acquired complementary superiority to each other. The taxa and function of horizontally transmitted bacteria often correspond with the requirements of the host, which implies that the horizontal transmission of bacteria between yaks and plateau pikas on the QTP has been determined by a selective process and has experienced strict filtration to meet the hosts’ requirements.

## Data Availability

The 16S rDNA data of fecal microbiota from plateau pikas and yaks can be freely retrieved from the NCBI Sequence Read Archive with project accession Nos. PRJNA699741.

## Supplementary Materials

The following are available online at https://www.mdpi.com/article/10.3390/microorganisms9091890/s1, Figure S1: Relative abundance of predicted gene of metagenome related to KEGG pathways; red box: based on host inherent OTUs without horizontal transmitted OTUs, blue box: based on total OTUs with the horizontal transmitted OTUs. (A) plateau pika, (B) yaks. Table S1: Sampling information of different groups. The PA represented the pikas in allopatry (enclosure); PS represented sympatric pikas with yaks; YS represented sympatric yaks with pikas; YA represented the yaks in allopatry (farm). Table S2: Phylum-level composition of gut microbiota across all samples. Table S3: Phylum-level composition of horizontally transmitted gut microbiota. Table S4: Genus-level composition of horizontally transmitted gut microbiota. Table S5: Enzyme produced by horizontally transmitted gut microbiota.
