# Supplementary material for: Sympatric Yaks and Plateau Pikas Promote Microbial Diversity and Similarity by the Mutual Utilization of Gut Microbiota
Source: Microorganisms. 2021 Sep 6;9(9):1890. doi: 10.3390/microorganisms9091890 (PMC8467723; doi:10.3390/microorganisms9091890)
Supplement: Supplementary file 1 [file microorganisms-09-01890-s001.zip › microorganisms-1330650-supplementary/Supplementary.pdf]

A

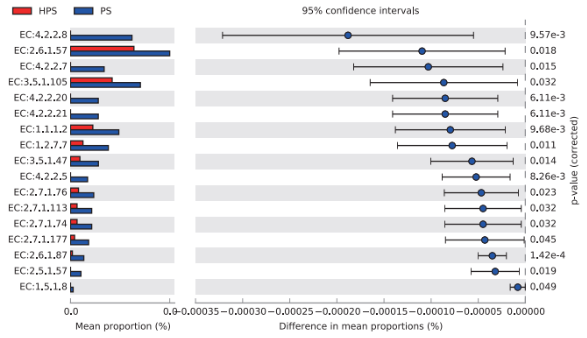

HPS
PS
95% confidence intervals
EC:4.2.2.8
EC:2.6.1.57
EC:4.2.2.7
EC:3.5.1.105
EC:4.2.2.20
EC:4.2.2.21
EC:1.1.1.2
EC:1.2.7.7
EC:3.5.1.47
EC:4.2.2.5
EC:2.7.1.76
EC:2.7.1.113
EC:2.7.1.74
EC:2.7.1.177
EC:2.6.1.87
EC:2.5.1.57
EC:1.5.1.8
9.57e-3
0.018
0.015
0.032
6.11e-3
6.11e-3
9.68e-3
0.011
0.014
8.26e-3
0.023
0.032
0.032
0.045
1.42e-4
0.019
0.049
p-value (corrected)
Mean proportion (%)
Difference in mean proportions (%)

B

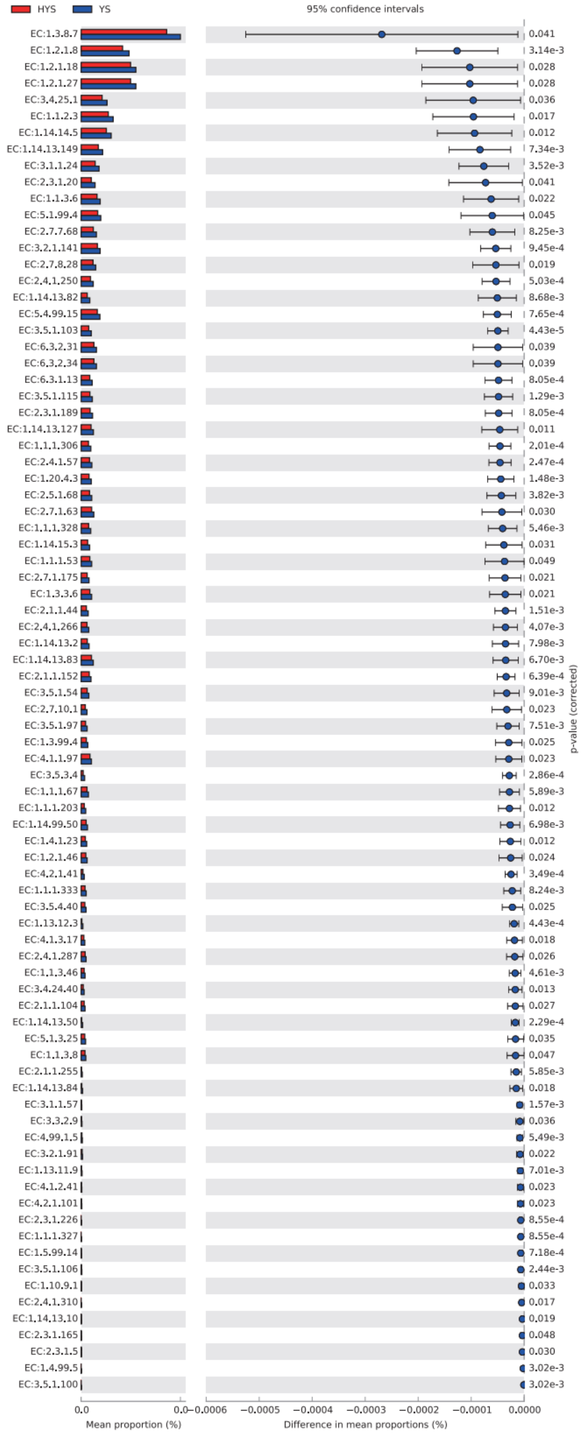

HYS
YS
95% confidence intervals
p-value (corrected)
Mean proportion (%)
Difference in mean proportions (%)

1

Fig. S1. Relative abundance of predicted gene of metagenome related to KEGG pathways; red box: based on host inherent OTUs without horizontal transmitted OTUs, blue box: based on total OTUs with the horizontal transmitted OTUs. (A) plateau pika, (B) yaks.

Sample type	Species	Lifestyle	Sample number
PS(pika in sympatry)	Plateau pika	Living in sympatry with yak	13
YS(yak in sympatry)	Yak	Living in sympatry with pika	10
PA(pika in allopatry)	Plateau pika	Living in enclosure (Isolated)	11
YA(yak in allopatry)	Yak	Living in farm (Isolated)	9
			Total: 43

Table S1. Sampling information of different groups. The PA represented the pikas in allopatry (enclosure); PS represented sympatric pikas with yaks; YS represented sympatric yaks with pikas; YA represented the yaks in allopatry (farm).

Table S2. Phylum-level composition of gut microbiota across all samples.

Table S3. Phylum-level composition of horizontally transmitted gut microbiota.

Table S4. Genus-level composition of horizontally transmitted gut microbiota.

Table S5. Enzyme produced by horizontally transmitted gut microbiota.
